# Bronchiectatic Actinomycosis with Osseous Metaplasia Masquerading as Lung Cancer

**DOI:** 10.5146/tjpath.2024.13407

**Published:** 2024-09-02

**Authors:** Archana Bhat, Manjunath J, Don Mascarenhas

**Affiliations:** Department of Pathology, Father Muller Medical College, Mangalore, India

**Keywords:** Diagnostics, Infection, Pneumonia, Haemoptysis, Consolidation

## Abstract

Bronchial involvement in pulmonary actinomycosis is rare and has been reported in the literature rarely. However, these reports describe endobronchial actinomycosis secondary to foreign body aspiration (for example, a fish bone). Our case did not have any history or clinical evidence suggesting foreign body aspiration, which makes it even more rare.

A 55-year-old woman presented with complaints of on and off haemoptysis and cough for three weeks. In view of the haemoptysis and consolidation seen on imaging, a bronchoalveolar lavage was done and sent for cytological assessment. Few atypical cells with nuclear hyperchromasia and prominent nucleoli were noted. In view of the persistent haemoptysis, worsening symptoms, and non-resolution of the consolidation despite antibiotics, and the finding of atypical cells, segmental resection was done. A final diagnosis of bronchiectatic actinomycosis with osseous metaplasia was given. The patient was started on prolonged antibiotics with good response and recovery.

Other risk factors associated with pulmonary actinomycosis include alcoholism, diabetes, haematological diseases, human immunodeficiency viral infection, use of immunosuppressants, and rarely chronic lung diseases, such as bronchiectasis. Our case had this rare association of bronchiectasis with bronchial actinomycosis.

Bronchiectatic actinomycosis is a rare infection and it can mimic several lung disorders like unresolving pneumonia, pulmonary tuberculosis, foreign body, and even lung tumours. The pathologists and clinicians should be aware of this entity and thus help in the early diagnosis and better management of patients with this disease.

## INTRODUCTION

Actinomycosis is a chronic and rare granulomatous disease caused by the filamentous anaerobic Gram-positive bacteria of the family Actinomycetaceae (genus Actinomyces) (1). Actinomycosis can affect any organ in the body. Pulmonary actinomycosis constitutes 15% of the total disease burden and is the third most common form of disease after cervicofacial and abdominopelvic forms (2). The pulmonary form is associated with concomitant respiratory disorders like emphysema, chronic bronchitis, bronchiectasis, and suppurative infections. Alcoholism, poor oral hygiene, dental disease, and facial or dental trauma are important risk factors (3). It is a rare infection with an occurrence of 1 in 3,00,000 people per year (3). Primary endobronchial actinomycosis is rare, and often associated with foreign body aspiration. It can mimic malignancy, tuberculosis, or nocardiosis, due to its continuous spread and progression, and tendency to form a cold abscess (4). A high level of clinical suspicion is needed for its diagnosis. We present a case of bronchiectatic actinomycosis without any evidence of foreign body aspiration, which closely mimicked malignancy.

## CASE REPORT

**Figure 1:**
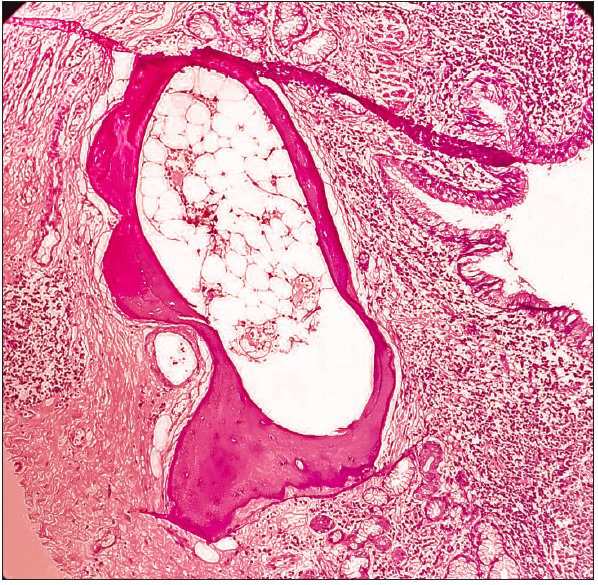
Haematoxylin and eosin stain, 10X, showing dilated bronchi lined by respiratory epithelium. Dense inflammation is seen in the wall along with osseous metaplasia.

**Figure 2:**
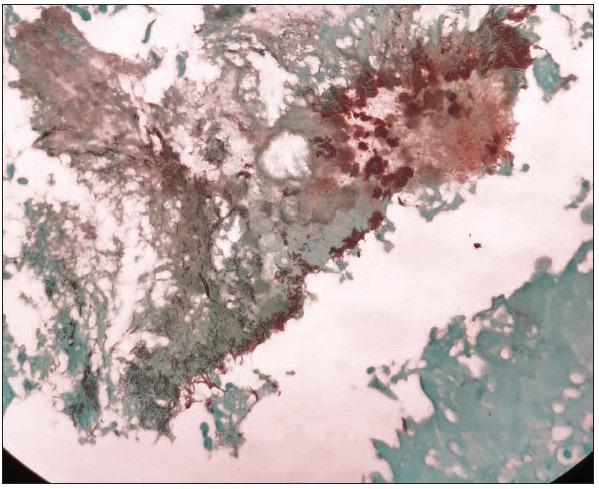
Gomori methenamine silver stain, 40X, showing silverpositive intraluminal radiating filamentous bacterial colonies.

A 55-year-old woman presented with complaints of on-and-off haemoptysis and cough for three weeks. There was no history of fever, vomiting, weight loss, or breathing difficulties. There was no history of any trauma. She had no known comorbidities. Her history revealed that she had undergone total abdominal hysterectomy with bilateral salphingo-oophrectomy with pelvic lymph node dissection for endometrial carcinoma twelve years ago. Her respiratory examination findings revealed bilateral air entry with coarse crepitations on the right side. The abdomen was soft and non-tender, and no abnormality was detected. Cardiovascular and central nervous system examination were within normal limits. In view of hemoptysis and consolidation on imaging, a bronchoalveolar lavage was done and sent for cytological assessment. Few atypical cells with nuclear hyperchromasia and prominent nucleoli were noted. Tuberculosis was ruled out. In view of the persistent hemoptysis, worsening symptoms, and non-resolution of consolidation despite antibiotics, and the finding of atypical cells, a segmental resection was done. The gross pathology revealed dilated bronchi and areas of congestion. No growth or necrosis was seen. Microscopy revealed dilated bronchi with inflammation and osseous metaplasia (Figure 1). Intraluminal radiating filamentous bacterial colonies were seen, which stained positive with the Gomori methenamine silver stain (Figure 2) and gram stain, and were negative with the modified acid-fast stain. A final diagnosis of bronchiectatic actinomycosis with osseous metaplasia was given. The patient was started on prolonged antibiotics with good response and recovery.

## DISCUSSION

Bronchial involvement in pulmonary actinomycosis is rare and has been reported in the literature rarely. However, these reports describe endobronchial actinomycosis secondary to foreign body aspiration (for example, a fish bone) (5). Our case did not have any history or clinical evidence suggesting foreign body aspiration, which makes it even more rare.

Other risk factors associated with pulmonary actinomycosis include alcoholism, diabetes, hematological diseases, human deficiency viral infection, use of immunosuppressants, and rarely chronic lung diseases, such as bronchiectasis (6). Our case had this rare association of bronchiectasis with bronchial actinomycosis.

The suspicion of pulmonary actinomycosis is commonly raised by radiological findings like interstitial or pleural thickening, endobronchial mass causing atelectasis, lobar or air-space consolidation, ground glass opacities, pleural effusion, hilar lymphadenopathy, or necrotic mass. However, the diagnosis remains challenging due to the absence of any radiological features that are specific to the disease (7).

The bronchoscopic features of endobronchial actinomycosis are non-specific and include wall thickening with partial or complete occlusion of bronchi or an exophytic mass or a submucosal mass lesion, at times with necrosis, mimicking tuberculosis, or lung cancer (8). Thus a wide range of differentials enter the list including tumours - both benign and malignant, infective causes, and miscellaneous causes. The benign tumours include namely pulmonary hamartoma, lipoma, carcinoid, and fibroepithelial polyp. The malignant tumours include neuroendocrine carcinoma, bronchogenic tumours, endobronchial metastasis, and mucoepidermoid carcinoma. The infective endobronchial lesions include endobronchial tuberculosis or nocardiosis, and miscellaneous causes include mucus plug or foreign body (9). Pulmonary actinomycosis can be easily confused with other diseases. This may lead to unnecessary surgeries (10).

The reactive pneumocytes or reactive bronchial epithelial cells can masquerade as malignant cells on bronchoalveolar lavage or bronchial brush smears, as it happened in our case. Histopathological examination and microbiological evaluation will help in arriving at a definitive diagnosis. Early diagnosis and treatment will help in preventing dangerous complications like rib fractures, cardiac involvement, or fatal pericarditis (9).

## CONCLUSION

Bronchiectatic actinomycosis is a rare infection and it can mimic several lung disorders like unresolving pneumonia, pulmonary tuberculosis, foreign body, and even lung tumours. Pathologists and clinicians should be aware of this entity and thus help in the early diagnosis and better management of patients with this disease.

## Conflict of Interest

The authors declare that they have no conflict of interest.
