# Correction: Bronchiectatic Actinomycosis with Osseous Metaplasia Masquerading as Lung Cancer

**DOI:** 10.5146/tjpath.2024.13407err

**Published:** 2024-09-02

**Authors:** Archana Bhat, Manjunath J, Don Mascarenhas

**Affiliations:** Department of Pathology, Father Muller Medical College, Mangalore, India; Department of Pulmonology, Father Muller Medical College, Mangalore, India

After the publication of the original article, the authors noticed an error in the departmental affiliation of one of the contributors, Don MASCARENHAS. The corrected version of the department is provided below, and the original article has been updated accordingly.

